# Strategic Self-Regulation in Groups: Collective Implementation Intentions Help Cooperate When Cooperation Is Called for

**DOI:** 10.3389/fpsyg.2020.561388

**Published:** 2020-11-24

**Authors:** J. Lukas Thürmer, Frank Wieber, Peter M. Gollwitzer

**Affiliations:** ^1^Department of Psychology, University of Konstanz, Konstanz, Germany; ^2^Department of Psychology, University of Salzburg, Salzburg, Austria; ^3^Institute of Health Sciences, Zurich University of Applied Sciences, Zurich, Switzerland; ^4^Department of Psychology, New York University, New York City, NY, United States; ^5^Institute of Psychology, Leuphana University of Lüneburg, Lüneburg, Germany

**Keywords:** collective implementation intentions, small group performance, self-regulation, cooperation, prisoners’ dilemma, motivation science

## Abstract

Groups need contributions that are personally costly to their members. Such cooperation is only adaptive when others cooperate as well, as unconditional cooperation may incur high costs to the individual. We argue that individuals can use *We*-if-then plans (collective implementation intentions, cIIs) to regulate their group-directed behavior strategically, helping them to cooperate selectively with group members in the situation planned for. In line with this prediction, a cII to consider group earnings increased cooperative decisions in a prisoners’ dilemma game when playing against another group member but not when playing against a stranger (i.e., non-group member). Moreover, cIIs to cooperate in the prisoners’ dilemma game did not increase cooperation in a structurally similar investment game that participants had not planned for. We discuss the role of collective planning in solving social dilemmas.

## Introduction

“*Aside from the moral issue, a man who does not show backbone acts unwisely. He invites the bestiality of the mob which is always ready to have its brutal fun but is afraid to stick out its neck when it knows that it will be resisted*” (Lewin, 1939/1997).

*Teamwork* implies cooperation; *groups* imply the pursuit of social goals; the collective good requires individual sacrifice. All these assumptions are common in research and the general public, indicating that individual cooperation and sacrifice are crucial to ensure group success (Kerr, 1983). At the same time, unconditional cooperation may leave the individual vulnerable to exploitation. As Lewin (1939/1997) indicates, strangers may take one’s cooperation for granted and exploit those who behave too agreeably. Even members of one’s own group may come to realize that they can free ride on the goodwill and effort of unconditionally cooperative members, thereby minimizing their own contributions to the group (Kerr, 1983). Because cooperation is beneficial in some contexts but detrimental in others, *conditional cooperation* is key to the welfare and performance of groups and their members.

However, how can groups ensure such conditional cooperation? Existing approaches for increasing cooperation commonly rely on direct interaction between members, thereby allowing members to punish free-riders (e.g., by pursuing a *tit-for-tat* strategy). Group contexts may often not afford the necessary information, as group members only sporadically learn about others’ decisions or the outcomes achieved by the entire group. We will argue that group members have to rely on their capacity to regulate their behavior adaptively. We also suggest that mere goals are insufficient and that group members will benefit from planning prospectively *when*, *where*, *how*, and *with whom* to cooperate, thereby using their individual self-regulation capability to steer group processes.

### Importance of Conditional Cooperation in Groups

To perform successfully, groups require the contributions of their members (Steiner, 1972; Hinsz et al., 1997; Hinsz and Ladbury, 2012), but group members can often enjoy the benefits of their group’s performance even if they do not contribute adequately (Kerr, 1983, 2013; Parks, 2020). When contributing to a group performance comes at a personal cost, a conflict arises between the group’s maximal performance and the individual’s temptation to engage in free-riding. These *social dilemmas* are difficult to resolve (Komorita and Parks, 1995; Weber et al., 2004) because it is tempting to follow selfish interests even when one has cooperative group goals (Sheldon and Fishbach, 2011). For instance, even when a team member has the goal to promote a joint project, it may be tempting to take the afternoon off.

Decisions in prisoners’ dilemma games (PDG) reveal whether group members maximize their individual or collective performance because they pose an explicit conflict between collective and individual outcomes (Dawes, 1980; Weber et al., 2004; Messick and Brewer, 2005): Two players choose between two options, and their outcomes depend on each other’s choices. One of the choices (usually termed *defect*) leads to greater individual profit than the choice (usually termed *cooperate*), regardless of the other player’s decision. However, mutual cooperation leads to better joint outcomes than mutual defection. The collectively rational choice is thus to cooperate. However, because the choice to defect leads to greater individual profits, it is tempting for the individual, and deciding in favor of one’s group (i.e., cooperate) requires self-control (Sheldon and Fishbach, 2011; Martinsson et al., 2012). Indeed, even when committed to the collective goal of securing common, long-term benefits, individuals often act selfishly and in line with their individual goal (Komorita and Parks, 1995). In short, it is difficult to prioritize the collective goal over the conflicting individual goal in social dilemma situations.

This conflict between individual and collective goals is even stronger in dilemma games with monetary incentives and when participants do not repeatedly interact (i.e., in *one-shot* games). When decision-dependent monetary incentives are offered, the task poses a real conflict to participants (Smith, 1976), and prioritizing group welfare on the spot becomes even more difficult than in hypothetical tasks. Similarly, in one-shot games, a competitor cannot reciprocate cooperation. Reciprocity (including punishment) is known to increase cooperation in iterated dilemma games (Axelrod and Hamilton, 1981; Fehr and Gächter, 2002) and allows for self-interested cooperation (e.g., favorable self-presentation; Danheiser and Graziano, 1982; maximizing monetary earnings; Kreps et al., 1982). Thus, as reciprocity is impossible in one-shot games; defection becomes the dominant strategy, as it yields greater payoffs for the individual regardless of the competitor’s choice (Camerer, 2003). Moreover, under anonymous conditions, self-presentation concerns are unlikely to operate. In sum, anonymous and incentivized one-shot dilemma games promote the individual goal to defect and make it difficult to prioritize the collective goal to cooperate.

Despite the importance of cooperation for group outcomes, unconditional cooperation is unwise. It is easy for free-riders to exploit the goodwill of those who always cooperate (Lewin, 1939), and it is therefore important to carefully choose whom to cooperate with – and when (Axelrod and Hamilton, 1981; Ohtsuki, 2018). Although such *conditional cooperation* is crucial, only approximately 50% of people follow this strategy, and this holds true even when full information on the other actors is available (Fischbacher et al., 2001). In group contexts, cooperation decreases considerably between members of different groups (Reinders Folmer et al., 2012). However, even within one’s group, one may agree to cooperate on certain tasks but not on others. Unconditional cooperation may invite free-riders to exploit the group, and it is thus important to stick to the cooperation one has agreed upon. However, how can groups put conditional cooperation into action?

### Regulating Goal Striving in Groups With Collective Implementation Intentions

At this point, one may argue that groups should set strong cooperative goals (Zander, 1971; Shaw, 1976; Karau and Williams, 1993) that refer to group outcomes (collective goals; e.g., “we want to make cooperative decisions;” Weldon and Weingart, 1993; Locke and Latham, 2006) to ensure group success. However, analogous to the individual level (Sheeran and Webb, 2016; Webb and Sheeran, 2006), a strong commitment to a group goal is not sufficient for group goal attainment (Wieber et al., 2012, 2013). In line with this reasoning, Seijts and Latham (2000) noted that “A social dilemma appears to be a boundary condition for the normally positive effect of group goal setting on group performance” (p. 104). Group goals are, therefore, not sufficient to ensure cooperation.

Collective implementation intention (cII) theory proposes that groups can resort to if–then plans to narrow this gap between cooperative intentions and cooperative actions (Thürmer et al., 2015a, 2017, 2020b). At the individual level, forming *implementation intentions* (IIs) that specify in advance when, where, and how to act in an if–then format (e.g., “And if *I* encounter situation y, then *I* will show the goal-directed response z!”; Gollwitzer, 1993, 1999, 2014) have been found to reliably facilitate goal striving and attainment in academic, health, and interpersonal domains (meta-analyses by Gollwitzer and Sheeran, 2006; Adriaanse et al., 2011; Webb et al., 2012; Bélanger-Gravel et al., 2013; Chen et al., 2015; Toli et al., 2016; Vilà et al., 2017; Silva et al., 2018; McWilliams et al., 2019; meta-analysis of meta-analyses by Keller et al., 2020). Building on this experimental research, cII theory proposes that groups can resort to traditional if–then plans (*I*–if–then plans) but also to *We*–if–then plans. Like individual IIs, such cIIs are if–then plans that specify when, where, and how to act toward a set goal. Different from IIs, cIIs refer to the group (e.g., “And if *we* encounter situation Y, then *we* will show response Z!”). A growing body of empirical evidence indicates that cIIs can indeed promote group goal attainment.

Initial studies observed cII effects in decision-making tasks, focusing on the integration of socially and temporally distributed information. In the first set of studies, a cII to review information enabled groups to review and integrate socially distributed information in hidden profile problems, leading to more informed decisions (Thürmer et al., 2015b). Moreover, a cII to take an observer’s perspective enabled groups to integrate emerging information in an escalation of commitment task, leading to more prudent investment decisions (Wieber et al., 2015a). These papers indicate that cIIs can promote group goal striving, much like IIs do at the individual level.

Recent research has started to investigate potential differences between cIIs and traditional IIs in groups, indicating that cIIs induce a group-focus (Thürmer et al., 2017). In one study, three group members jointly performed a physical persistence task (Bray, 2004) that requires each member’s contribution (Kerr and Hertel, 2011). After performing a baseline round of the task, groups either formed a cII or an II or received respective control instructions (i.e., collective vs. individual goal intentions). Forming cIIs and IIs both improved performance in comparison with the respective control groups without an if–then plan, but these effects seemed to rely on different processes. Groups that had formed a cII communicated more with each other than did II groups. Moreover, cII groups referred more to the group (first-person plural pronouns were used; cf. Pennebaker et al., 2003), but the II group members referred more to themselves (first-person singular pronouns were used). A second experimental study confirmed the causal role of the group-focus: the cII led to better performance when participants were encouraged to communicate, and the II led to better performance when participants were prevented from communicating. In sum, an increased group focus qualifies as a process leading to cII effects but not II effects, suggesting that cIIs should help prioritize group outcomes over individual outcomes in social dilemmas.

All these existing studies leave open whether cII effects are specific to one’s group. As outlined earlier, such conditional effectiveness would be an important precondition for cIIs to promote group performance safely. At the individual level, if–then plans heighten the accessibility of the situation specified in the if-part (Wieber and Sassenberg, 2006). One may therefore argue that referring to a collective in an if–then plan (i.e., forming a cII) heightens the accessibility of “other-related” concepts (Wong and Hong, 2005; Drouvelis et al., 2015), thereby increasing cooperation regardless of whether one encounters someone who is a group member or not. As discussed earlier, such unconditional cooperation would leave group members vulnerable to exploitation by strangers and encourage free-riding of malicious group members.

In contrast, the theory of cIIs predicts that cIIs *strategically* increase cooperation, that is, within the group and in the situation planned for. At the individual level, traditional IIs are dependent on the goal that they are formed for (Gollwitzer et al., 2008; Legrand et al., 2017), and cII theory, therefore, assumes that *We*–if–then pans support collective goals held by one’s group. Identification with one’s group is a prerequisite for holding collective goals (van Knippenberg and Ellemers, 2003), implying that the group goal to cooperate should only be activated when facing a group member but not when facing a stranger. Consequently, a cII to focus on the group outcome should increase cooperation when facing a group member but not when facing a stranger, thus being specific to one’s group.

A related open question concerns whether cII effects are specific to the situation included in the plan. Groups of defectors take advantage of cooperative members, and it would, therefore, also be important to cooperate strategically within the group (Efferson et al., 2016). Such conditional cooperation would then deter free-riders within the group and ensure good group performance. One could argue that since cIIs link the group (included in the situation) to a cooperative response, this link should lead to unconditional cooperation within the group. However, IIs at the individual level are known to facilitate only the preplanned responses (Parks-Stamm et al., 2007; Masicampo and Baumeister, 2012). Accordingly, *We*–if–then plans should facilitate the preplanned response in the specified situation but not in other situations, even when these other situations are structurally similar.

## Present Research

Building on prior research on cIIs, the current study investigated whether *We*–if–then plans can promote cooperation in social dilemma situations and whether the expected cII effects are specific to both (a) one’s group and (b) the preplanned situation. To test these assumptions, we developed an airline pricing game (based on Sheldon and Fishbach, 2011) where group members could choose between cooperation (an individually unprofitable and collectively profitable choice) and competition (an individually profitable choice that is collectively unprofitable). The task thus posed a social dilemma. Group members either formed a cII to focus on the group outcome, an II to focus on the individual outcome, or formed a control if–then plan that referred to neither the group outcome nor the individual outcome. Participants then played against alleged other group members and against non-group members. To preclude the operation of ulterior motives for cooperation (e.g., favorable self-presentation; Danheiser and Graziano, 1982; maximizing monetary earnings; Kreps et al., 1982), participants did not receive feedback on theirs or their partner’s decisions (i.e., multiple one-shot games), and their identity was completely anonymous. Lastly, participants played a structurally similar game that they had not planned for. We expected cIIs to increase costly cooperation within one’s group but not with non-group members and in the situation planned for but not in similar situations that participants had not planned for.

## Materials and Methods

### Participants and Design

We collected a convenience sample of 134 University of Konstanz students (80 female) with a mean age of 20.90 years (*SD* = 2.58). Participants received a decision-dependent monetary incentive (see *PDG* Task); participants earned 4.51€ on average (*SD* = 2.20). The experiment followed a 2 within (Competitor: alliance member vs. non-alliance member) × 3 between (II: control vs. II vs. cII) mixed factorial design. Power analyses indicated that the achieved sample size was sufficient to detect a medium-sized interaction effect (1 – B = 0.99). We moreover added a 2 between control factor (Explicitly Assigned Goal: individual vs. collective) to explore whether an explicit individual or collective goal would already impact dilemma decisions; the control factor was fully crossed with the II factor.

### Procedure and Manipulation

After giving informed consent, all participants learned that the study concerned business decision-making and that they would be paid according to the decisions made: at the end of the experiment, the decision from one randomly chosen trial was matched with the decision of another randomly selected previous participant to determine their decision-payoff. There was no deception concerning the payoff (for the first participants, actual decisions from voluntary pre-testers blind to the hypotheses were used).

Participants all received the individual code name *International Airline* and learned that they were part of the group *Flugallianz* (see section “Materials”). To test whether explicitly assigned goals are sufficient to increase cooperation, half of the participants were asked to set the individual goal “I want to maximize International Airline’s revenue” during the instructions for the non-alliance task (see section “Materials”); the other half of the participants were asked to set the explicit collective goal “We want to maximize *Flugallianz*’s revenue” during the instructions for the alliance task. To make sure that all participants took equal time to think about the task at hand, participants who did not receive a respectively assigned goal were instructed to pay close attention to how both players’ decisions influence each other.

Before working on the decision trials, participants received a printed training sheet to manipulate the II factor. Participants in the cII condition made the collective if–then plan “And when we are about to make our decision, then we will make sure that *Flugallianz* receives the highest payoff!” Participants in the II condition set the individual if–then plan “And when I am about to make my decision, then I will make sure that International Airline receives the highest payoff!” To minimize the differences between conditions, control participants received the neutral control plan “When a decision screen appears, then a decision has to be made.” This plan was also phrased in an if–then format and referred to making decisions but referred to neither the individual nor the group.

Eight alliance trials followed (see *PDG* Task in section “Materials”). Payoff matrices were used once with each competitor and presented in a fully randomized order. After a 30-s break, participants worked on eight trials of the no-alliance task, supposedly against two non-group members (i.e., the non-alliance members *Fly Jet* and *City Connect*). To discourage cooperation for individual benefit (e.g., maximizing profits with a tit-for-tat strategy or strategic self-presentation), participants did not learn about other participants’ decisions until after the experiment. Cooperation thus mainly benefited the group.

To test whether the expected increases in cooperation through cIIs spill over to other tasks, participants then played the hypothetical investment task against a group member (*Metropolis Airways*) and a non-group member (*Fly Jet*). Finally, participants answered three questionnaires concerning their commitment to the explicitly set goal (seven items, e.g., “This is a goal to shoot for,” 1: *not at all* to 7: *completely*, *Cronbach’s* α = 0.70, Klein et al., 2001), plan commitment (e.g., “I would like to fulfill my plan,” *Cronbach’s* α = 0.86), and group identification (e.g., “It is important to me to belong to my group,” *Cronbach’s* α = 0.83).

### Materials

We developed a PDG task (based on Sheldon and Fishbach, 2011) that included a group membership to allow for the collective goal to cooperate. We conducted a pilot study to confirm this assumption. Lastly, we used an investment task (adapted from Fischbacher et al., 2001).

### Prisoners’ Dilemma Games Task

**FIGURE 1:**
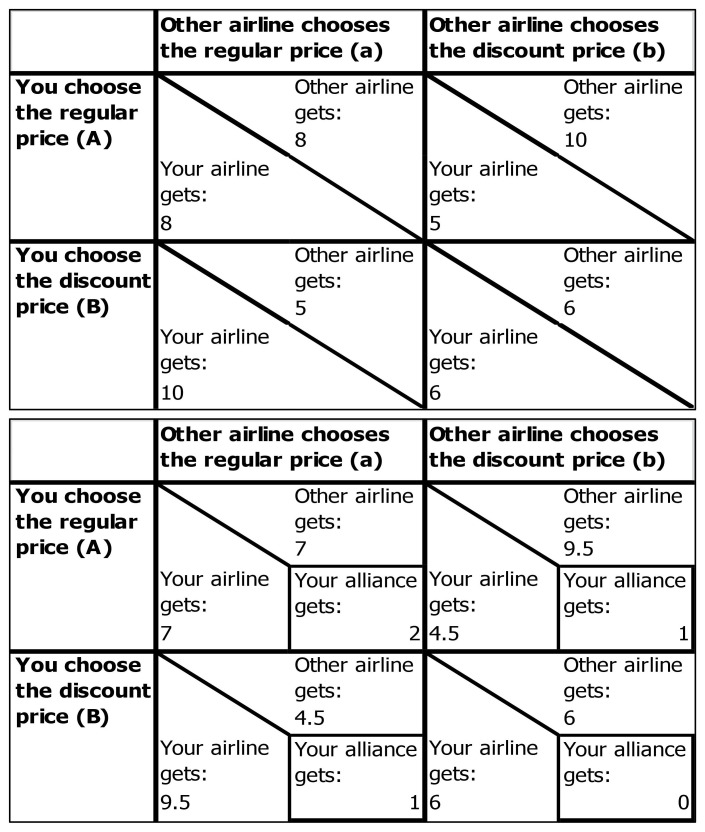
**(A)** No-alliance (non-group member) payoff matrix. In this prisoners’ dilemma task, the regular price (A) can be considered to be the cooperative decision due to the higher joint payoff (A,a > B,b); the discount price (B) can be considered to be the defect decision due to the higher individual payoff (B,a > A,a; B,b > A,b). **(B)** Alliance payoff matrix. Alliance payoffs are paid to the players 50/50, and this matrix thus leads to identical payoffs to the no-alliance matrix. However, alliance payoffs visualize the difference in joint payoffs according to each combination of decisions.

The task was introduced as an airline pricing game, and participants learned that they would be paid according to one of their decisions (see section “Procedure and Manipulation”). To prevent experimenter demand, the instructions clearly stated participants were free to decide on either option. It was explained that participants had to take on different roles (i.e., representing different airlines) to provide a meaningful competition context. Actually, all participants assumed the role of the *International Airline* CEO and were asked to remember this airline well, as it was their individual codename for the study and then prompted to type it (free recall). Participants then learned that they were to decide on the pricing of their tickets for different routes of their airline services (adapted from Sheldon and Fishbach, 2011): they could either choose standard pricing (cooperate) or discount pricing (defect). However, each route was also serviced by another airline that chose between the two pricing options, and the outcome of both airlines’ decisions influenced each other in a PDG fashion. An example payoff-matrix was provided (see Figure 1A) and explained in detail. Four questions followed to check whether participants understood the situations of mutual cooperation, mutual competition, and one player competing while the other cooperates (e.g., “How much do you earn when both choose standard pricing?/How much do you earn when both choose discount pricing?/How much do you earn when you choose discount pricing and the other airline chooses standard pricing?/How much do you earn when you choose standard pricing and the other airline chooses discount pricing?”). Participants with wrong answers were prompted to correct them; if needed, the experimenter reiterated the instructions. All participants could thus be expected to have experienced the individual temptation to defect.

Next, the group (alliance) was introduced: *International Airline*, *Air Oceanea*, *and Metropolis Airways* founded the *Flugallianz* to market residual tickets. Participants were informed that the alliance was important to the task, asked to memorize the alliance name, and type it (free recall). An example slide with the alliance’s situation was presented (see Figure 1B) and explained thoroughly. It was emphasized that the alliance-revenue for each connection would be divided between the two airlines servicing the respective route 50/50. When constructing the payoff matrices, we subtracted equal amounts from each player to create the alliance payoff. The actual payoff for individual and alliance trials was thus the same (see “Payoff-Matrices”). Again, participants had to respond to three questions correctly before they could continue with the experiment (“How much do you earn directly when both choose standard pricing? How much do you receive from the Alliance when both choose standard pricing? How much do you receive from the Alliance when both choose discount pricing?”). All participants could thus be expected to have formed the collective goal to cooperate through the instructions (see “Pilot Study”).

### Payoff-Matrices

We constructed four payoff-matrices: Cooperation-cooperation payoffs (a, A) ranged from 4 to 7; this difference was deemed sufficiently small to prevent high-stakes effects (Burton-Chellew and West, 2012). Differences to the other payoffs were held constant across payoff-matrices (for the player: B, a +2, A, b −3, B, b −2) to keep the temptation to defect constant (Smith, 1976). We constructed Alliance matrices by subtracting equal amounts from both competitors in the respective field of the payoff matrix. As the alliance payoffs were divided equally between both airlines servicing the route (50/50), this left the payoff unchanged. Moreover, to prevent effects from making the same decision repeatedly, for each decision, two fictitious three-letter airport codes (e.g., STB-LMT) indicated a different flight route.

### Pilot Study

We ran a pilot study to ensure that the alliance task actually activated a cooperative group goal. The pilot thus followed a 2 (Competitor: alliance vs. non-alliance) within the design. Twelve students from the University of Konstanz (4 female, mean age = 22.83 years, *SD* = 2.79) participated in return for decision-contingent payment (see “Procedure and Manipulation”) and earned 3.29€ on average (*SD* = 0.95). After playing the PDG task (see *PDG* Task), participants responded to seven items designed to measure group identification (see earlier discussion, *Cronbach’s* α = 0.88). On average, participants cooperated less than they defected (*M* = 4.17 out of 16 trials, *SD* = 3.13). This indicates that the task posed a strong temptation to defect. To test whether group membership affected participants’ pricing decisions, the cooperation score was entered into a repeated-measure ANOVA with Competitor (group member vs. non-group member) as within-factor. As expected, participants cooperated more when faced with a group member (*M* = 2.75 out of 8 trials, *SD* = 2.30) than when faced with a non-group member (*M* = 1.42 out of 8 trials, *SD* = 1.38), *F*(1, 11) = 4.63, *p* = 0.05, part. η^2^ = 0.30. In line with this result, participants reported medium identification with the *Flugallianz*, *M* = 4.29, *SD* = 1.27. Together, the findings suggest that our alliance task successfully activated the collective goal to cooperate.

### Hypothetical Investment Task

We adapted an investment task from Fischbacher et al. (2001) as a second task, which poses a conflict between individual and group outcomes but that participants had not planned for. Each participant learned that they had $10,000 that could be invested in a common project account with another player or be kept in one’s own account. The other player would also have this choice. Money in one’s own account could be kept. All the contributions to the common project account would be added, and each airline would receive 75% of the total amount. Participants indicated in steps of $1,000 how much of their $10,000 they would like to transfer to the project account (i.e., $0, $1,000, $2,000, and so forth). Investing was thus a good opportunity to make money if both airlines contributed but required trusting the other player to contribute equally. Importantly, although this game is structurally equivalent to the dilemma game played in the main experiment, it confronts players with a situation they had not planned for. It is thus well suited to examine whether the cII led to generalized or situation-specific cooperation.

### Dependent Measures

Dependent measures were the number of cooperative decisions (i.e., number of trials where standard pricing was chosen) in alliance and non-alliance trials.

## Results

Unless indicated otherwise, we analyzed the data with a mixed ANOVA with Competitor (group member vs. non-group member) as a repeated factor and II (control vs. II vs. cII) and Explicitly Assigned Goal (individual vs. collective) as between factors.

### Manipulation Checks

Participants reported medium group identification, *M* = 4.57, *SD* = 1.06, commitment to their plan, *M* = 3.56, *SD* = 0.94, and commitment to their explicit goal, *M* = 4.86, *SD* = 0.74. Importantly, goal commitment did not differ between the explicit individual and the explicit collective goal condition, *F*(2, 128) = 1.07, *p* = .30, part. η^2^ < 0.01. This suggests that the current task allows for holding both individual and collective goals. Unexpectedly, participants with the control intention (unrelated, neutral if–then plan) reported more commitment to their assigned individual or collective goal than participants in the II and the cII conditions, *F*(2, 128) = 3.21, *p* = .04, part. η^2^ = 0.05. However, including goal commitment as a covariate did not change the results reported. No other main or interaction effects were observed for any of the control variables, *F*s < 2, *p*s > 0.15.

### Main Analyses

**FIGURE 2:**
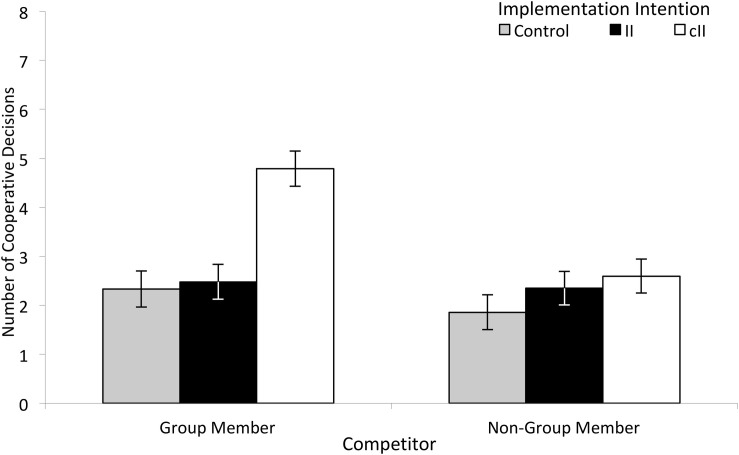
Number of cooperative decisions in the prisoners’ dilemma task by cII condition and competitor. Error bars represent standard errors.

Our first prediction stated that cII effects on cooperation are limited to members of one’s group. To investigate whether cIIs can help group members to keep their individual temptations in check, we analyzed the number of cooperative choices. We argued that cIIs support collective goal striving and thus should only increase cooperation when facing a group member. In line with this reasoning, the expected Competitor × II interaction was significant, *F*(2, 128) = 9.60, *p* < .01, part. η^2^ = 0.13: when competing against a group member, cIIs led to more cooperation, *M* = 4.80, *SD* = 2.67 (out of eight trials, Figure 2), than both IIs, *M* = 2.48, *SD* = 2.26, *p* < .01, and control instructions, *M* = 2.33, *SD* = 2.33, *p* < .01. As expected, there was no difference between conditions when competing against a non-group member, *M*_control_ = 1.86, *SD* = 1.95, *M*_II_ = 2.35, *SD* = 2.29, *M*_cII_ = 2.60, *SD* = 2.63, *p*s > .14. Thus, the cII did not blindly support cooperation but only when it served one’s group goal.

One might argue that a cooperative goal is sufficient to overcome a detrimental individual goal. However, neither a main effect of Explicitly Assigned Goal, *F*(2, 128) = 0.23, *p* = .64, nor a Competitor × Explicitly Assigned Goal interaction was observed, *F*(2, 128) = 2.64, *p* = .11 (see Table 1, for means and standard deviations). Accordingly, set goals to act in the interest of the group did not prevent selfish decisions; achieving cooperation required collective if–then plans.

**TABLE 1:** Cooperation with group members and strangers by implementation intention and explicit goal.

	**Explicit goal**					
	**Individual**			**Collective**		
**Measure**	**Control**	**II**	**cII**	**Control**	**II**	**cII**
Mean number of cooperative decisions with group members (out of 8)	2.09 (2.14)	2.48 (2.25)	4.27 (2.81)	2.57 (2.36)	2.48 (2.31)	5.30 (2.48)
Mean number of cooperative decisions with non-group members (out of 8)	1.91 (2.09)	2.78 (2.15)	2.36 (2.50)	1.81 (1.83)	1.91 (2.39)	2.83 (2.79)

*Standard deviations are in parentheses.*

As the II factor and the Explicitly Assigned Goal factor were crossed, we were also able to explore the effectiveness of cIIs when facing assigned antagonistic goals. The reported Competitor × II interaction was not qualified by a Competitor × II × Explicitly Assigned Goal interaction, *F*(2, 128) = 0.06, *p* = .94. Apparently, the cII was even effective in dealing with an assigned antagonistic individual goal. This observation is in line with (Sheeran et al., 2005, Study 2) finding that primed goals furnished with IIs are even attained in the face of assigned antagonistic goals.

Our second prediction stated that cII effects on cooperation are limited to the critical situation specified in the plan. Although cIIs do not lead to general cooperation with people outside of one’s group, they might lead to cooperation in general within one’s group; in other words, their enactment might not depend on the specified situation. General cooperation within one’s group should then spillover, for instance, to the investment task participants played at the end of the experiment. In contrast, we predicted that cII effects would be specific to the situation included in the plan. Analyzing the amount invested in this second game showed that participants generally invested more into a project with a group member, *M* = 6.74 (out of 10), *SD* = 2.82, than with a non-group member, *M* = 5.84, *SD* = 3.37, *F*(2, 127) = 13.47, *p* = .01, part. η^2^ = 0.09 (one participant failed to complete this task and was consequently excluded from this analysis). However, neither a main effect of II, *F*(2, 127) = 0.47, *p* = .63, nor an II × Competitor interaction emerged, *F*(2, 127) = 1.63, *p* = .20. This indicates that the cII did not generally increase participants’ cooperation, not even with group members; instead, cIIs specifically increased cooperation in the situation planned for.

In sum, the cII helped to resist an individual temptation for the sake of one’s group, even if this individual temptation was supported by an explicitly set goal. However, the cII did not increase cooperation when the collective goal was not activated (i.e., when facing non-group members) or in another situation not specified in the if-part of the plan (i.e., in an unrelated investment task). This pattern of results suggests that cIIs selectively support the collective goal they are formed for, even when this collective goal conflicts with the individual’s selfish goals. However, the group context planned for and the situation specified in the if-part of a cII need to be in place; these prerequisites thus qualify as moderators of cII effects.

## Discussion

Group performance and human success depend on cooperation. At the same time, unconditional cooperation leaves group members vulnerable to exploitation, may encourage others to take a free ride, and ultimately diminish group outcomes. We argued that group goals are insufficient to increase such conditional cooperation when the collective goals and the individual goals are in conflict (i.e., in a social dilemma situation) and that groups need *We*–if–then plans (cIIs; cIIs). Indeed, cIIs increased cooperation with group members in a PDG. Decisions were anonymous and incentivized, providing participants with the opportunity and incentives to defect. Cooperation was thus costly to the individual but highly beneficial to the group. In line with our predictions, we observed that cIIs induced conditional cooperation, that is, cooperation specific to (a) one’s group and (b) the situation planned for. Such conditional cooperation can be expected to maximize group performance in the long run.

These findings are key to the theory of cIIs. Conditional cooperation is crucial to ensure the effective functioning of groups, and cIIs promote such adaptive group behavior. Regarding the processes underlying cII effects, the current research indicates that the group context and the situation specified qualify as moderators. *We–*if*–*then plans thus do not turn group members into “collectivist robots” but promote adaptive and intentional group cooperation.

### Implications for Cooperation in Groups

We investigated cooperation in the context of a social dilemma with considerable incentives for individuals to defect. In line with past research (Locke and Latham, 2006), goals were not sufficient to ensure cooperation in this situation; instead, collective if–then plans were needed. Importantly, these plans increased cooperation selectively, thus not making the individual group member vulnerable to exploitation. This indicates that cIIs to cooperate are safe to apply in group settings, at least when formulated diligently (i.e., specifying one’s group and the prospective situation).

One may argue that cooperation will not always be the key to group success in dilemma situations. In fact, market structures may ensure that competition also yields beneficial collective outcomes (Maciejovsky and Budescu, 2007, 2013). Contrary to these findings, a recent study observed that competition and cooperation both increased effort early on during team tenure, but only cooperation had a positive effect later on (Hertel et al., 2018). Cooperative and competitive processes may thus lead to beneficial group outcomes. Support for this line of reasoning comes from classic research showing that purely individual motives, such as earning money in repeated interactions (Kreps et al., 1982) or gaining reputation where decisions are public (Danheiser and Graziano, 1982), may drive cooperation. Given that if–then planning is effective at the individual level and at the group level, it would be interesting to explore how plans across social levels can promote these different processes.

Teams perform many important tasks in organizations, and cooperation is key to team performance (Van Lange et al., 2013). Increasing cooperation by forming cIIs could thus be highly beneficial in work contexts (Thürmer et al., 2015a; Gagné, 2018). Although limited data is available on this question (Lehmann et al., 2019), research on social dilemmas and social loafing has proven highly influential in organizational teams (Stouten and Liden, 2020), and II effects have been observed in organizational contexts, such as adherence to new workplace regulations (Holland et al., 2006) and making entrepreneurial decisions (Adam and Fayolle, 2015; van Gelderen et al., 2017). It is, therefore, likely that cII effects on cooperation generalize to organizational settings (Thürmer et al., 2015a).

### Implications for Collective Action Control

The present research informs classic and current debates on how groups attain their goals. First, our research provides an empirical demonstration that committing to group goals may indeed be insufficient to ensure positive group outcomes (collective intention-behavior gap; Wieber et al., 2012, 2013). Although adopting explicit individual or collective goals had no effect on cooperation in our experiment, if–then plans significantly increased rates of goal attainment. This observation is in line with classic individual-level research (Gollwitzer, 1993, 1999, 2014) and recent group-level advances (Thürmer et al., 2015a, 2020b). In contrast to cIIs, IIs had no effects on cooperation. However, the IIs used in the current research focused on individual outcomes instead of group outcomes. Building on group-level goal setting research (Kleingeld et al., 2011), the pertinent next step would be to investigate whether and how classic IIs focusing on group outcomes can increase conditional cooperation.

Regarding the processes underlying cII effects, the present research highlights two important moderators. The specified situation emerged as a well-known moderator of if–then planning effects (Parks-Stamm et al., 2007); additionally, group membership emerged as a unique moderator of cII effects. This finding is in line with the goal-dependency of if–then plan effects (Sheeran et al., 2005), as group membership is a prerequisite for the commitment to group goals (van Knippenberg and Ellemers, 2003). Recent research moreover indicates that cIIs are particularly effective when using an if–then format (Thürmer et al., 2020a). Understanding these processes underlying cII effects will help maximize their effects across settings (Gollwitzer et al., 2010).

Finally, our research indicates that collective if–then plans help individuals overcome the temptation to follow their immediate self-interest. Future research should investigate whether a cooperative personality qualifies as a moderator of this effect. There is a substantial interindividual variation in whether people choose to cooperate (Parks, 2015, 2020), and these differences may have a key impact on teamwork (Graziano et al., 1997). Individual-level research indicates that if–then planning can protect people from acting on their unwanted personality traits (Hudson and Fraley, 2015). Moving toward a full situational analysis (Kelley and Thibaut, 1978; Kelley et al., 2003), exploring the interplay between personality and *We*–if–then planning is a highly fruitful direction for future research.

### Limitations and Future Directions

There are three limitations of the present research that warrant discussion. First, one may argue that gains in collective performance were relatively easy to achieve in the current task, as cooperation rates were generally quite low. However, cII effects have already been observed under conditions where collective effort is high (Thürmer et al., 2017) and in tasks with incentivized group performance (Thürmer et al., 2015b). It is thus unlikely that cIIs only improve group performance when groups perform poorly. Related to this, group interaction in the present study was delayed, such that there was no opportunity for reciprocity or face-to-face interaction. Both reciprocity and face-to-face interaction have a great impact on behavior in groups, leading to the question whether cIIs would also increase cooperation in such interactive settings. However, cII effects have already been demonstrated in face-to-face interactions (e.g., Thürmer et al., 2015b, 2017; Wieber et al., 2015a), indicating that cIIs could increase cooperation in interactive settings.

Second, one may argue that the present study used but one type of task and that task type is a crucial (yet often neglected) moderator in group research (Kerr, 2017). Past research has, however, observed cII effects in a wide range of tasks (Thürmer et al., 2020b). Still, a remaining question in this regard is which neural processes are underlying cII effects. Past research has illuminated the physiological processes underlying if–then planning in individuals (Wieber et al., 2015b; Wolff et al., 2018) and spontaneous action planning at the dyadic level (Kourtis et al., 2019). Combining both streams of research, ideally using neurological measures during performance tasks (Wolff et al., 2019), would help to understand further cII effects and their processes at the group level.

Third, we only investigated an intragroup setting and did not specify the group membership of non-group members. Intergroup settings may pose particular hindrances to successful self-regulation, such as the experience of negative emotions (Niedenthal and Brauer, 2012) or the ascription of negative intentions (Hornsey and Esposo, 2009), leading to costly defensive responses (Thürmer and McCrea, 2018, in-principal acceptance; Thürmer et al., 2019). These responses are hard to change in intergroup contexts. One promising exception is the use of compelling narratives that describe the flow of actions in context and thereby help focus on a new reality (Murrar and Brauer, 2019). IIs can help change the expression of negative stereotypes (Stewart and Payne, 2008; Mendoza et al., 2010), and our research suggests that *We*–if–then planning may help change (collectively) costly behavior. Future research should thus test whether combining convincing narratives with cIIs helps further reduce intergroup animosities.

## Conclusion

Cooperation ensures group success, but it also leaves people vulnerable to exploitation. The present research demonstrates that cIIs can help solve this dilemma. People with *We*–if–then plans cooperate when cooperation is called for, with the right people and in the right situation.

## Data Availability Statement

The raw data supporting the conclusions of this article will be made available by the authors, without undue reservation, to any qualified researcher presenting a legitemate interest.

## Ethics Statement

The study involving human participants was approved by the University of Konstanz. The participants provided their written informed consent to participate in this study.

## Author Contributions

JLT, FW, and PG jointly designed the studies and revised the manuscript. JLT and FW supervised data collection and analyzed the data. JLT prepared a first draft. This research was part of JLT’s doctoral dissertation. All authors contributed to the article and approved the submitted version.

## Conflict of Interest

The authors declare that the research was conducted in the absence of any commercial or financial relationships that could be construed as a potential conflict of interest.
